# Uncovering the Residual Electrolyte Quantity in Recycled Battery Black Mass via Liquid Chromatography Tandem Mass Spectrometry and Ion Chromatography‐Conductivity Detection

**DOI:** 10.1002/cssc.202501158

**Published:** 2025-08-18

**Authors:** Jakob Michael Hesper, Simon Weigel, Jan Henrik Hintemann, Jaroslav Minář, Martin Winter, Simon Wiemers‐Meyer, Sascha Nowak

**Affiliations:** ^1^ MEET Battery Research Center University of Münster Corrensstraße 46 48149 Münster Germany; ^2^ Königswarter & Ebell Chemische Fabrik GmbH Im Ennepetal 19‐21 58135 Hagen Germany; ^3^ Helmholtz‐Institute Münster, IMD‐4 Forschungszentrum Jülich Corrensstraße 46 48149 Münster Germany

**Keywords:** analytical methods, batteries, black mass, electrolyte residues, extraction methods

## Abstract

A workflow for the quantification of electrolyte residues, including linear and cyclic carbonates, conducting salt and selected degradation products from shredded lithium ion battery material black mass, is developed. Therefore, a liquid chromatography method hyphenated to a tandem mass spectrometer is set up which is capable of separating and reliably quantifying standard organic electrolyte compounds showing low limits of quantification and detection. For the quantification of ionic species, ion chromatography with a conductivity detector is used. The combination of data sets shows that up to 5.5 wt% of organic compounds and 2.46 wt% of ionic species are extracted from the black mass with ethylene carbonate and hexafluorophosphate (PF_6_^−^) being the most prominent species. The use of protic solvents, such as water and methanol, results in degradation reactions forming ethylene glycol and fluoride (F^−^) respectively. Three different extraction methods are evaluated for their applicability in a quantitative analysis setup. The results demonstrate that shake extraction using the aprotic acetonitrile is the most suitable sample preparation technique with over 90% extraction after a single cycle without introducing solvolysis nor thermal degradation.

## Graphical abstract

A workflow for the quantification of electrolyte residues, including linear and cyclic carbonates, conducting salt and selected degradation products from shredded lithium ion battery material black mass, is developed. The results demonstrate that shake extraction using the aprotic acetonitrile is the most suitable sample preparation technique with over 90% extraction after a single cycle without introducing solvolysis nor thermal degradation.

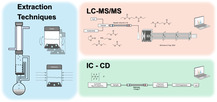

## 1 Introduction

To establish an independent and sustainable battery value chain, a closed‐loop recycling strategy is essential.^[^ ^1^, ^2^ ^]^ Recycling end‐of‐life lithium ion battery (LIB) cells is also crucial to prevent these cells from entering landfills, where harmful substances could be released into the environment, such as fluorine (F), nickel (Ni), cobalt (Co), and organic compounds originating from electrolyte components and its degradation products.^[^ ^3^, ^4^ ^]^ Additionally, understanding the composition of LIB recycling material is vital, as these contaminants can damage business infrastructure, hinder recycling processes, and potential health risks to the environment and employees.^[^ ^5^ ^]^

For hydrometallurgical LIB recycling strategies, the recycling material is typically crushed and mechanically sorted to gain an active material enriched material mix. This mix is called black mass. The analysis of the elemental composition of such black mass is well described using different techniques.^[^ ^6^ ^]^ Even fast screening methods which can be used for quality control, as described by Dommaschk et al. using atomic absorption spectroscopy, are already developed.^[^ ^6^, ^7^ ^]^ However, the focus of this work is set on the quantification of the electrolyte compounds which can be detected in black mass. Typical liquid LIB electrolyte consists of a mixture of linear and cyclic organic carbonates with a dissolved conducting salt (and additives).^[^ ^8^ ^]^ So far, numerous studies have focused on analytical methods tackling the identification of electrolyte compounds and degradation products in LIBs.^[^ ^9^, ^10^, ^11^, ^12^ ^]^ Several of these methods include rather demanding sample preparation as working under argon or dry room conditions. After cell opening, several steps of centrifugation and dilution are performed before measurement. of the extracted analyte can be performed. These extracts typically can be analyzed via gas chromatography (GC), liquid chromatography (LC), or ion chromatography (IC) hyphenated to flame ionization, mass spectrometry (MS), or conductivity detectors, respectively.^[^ ^6^ ^]^ These analytical methods are capable of performing in depth studies regarding identification of the main compounds of the electrolyte as well as degradation products to very low quantities.^[^ ^13^, ^14^ ^]^ Yet, one has to be careful when performing quantitative analysis, since not only the measurement of the extracted electrolyte has to be taken into account but also the extraction from the cell material has to be done quantitively regarding black mass, even such semiquantitative techniques are not yet developed.^[^ ^5^ ^]^ During the recycling process battery cells are deactivated, opened, shredded, and the materials mechanically separated.^[^ ^1^ ^]^ During the shredding process electrolyte is partially evaporated due to heat generation by the shredding process, intentional heating or by shredding using reduced pressures. Nevertheless, it was shown by Peschel et al. that commercial black mass still contains electrolyte and electrolyte decomposition products.^[^ ^6^ ^]^ Unfortunately, quantification of the electrolyte residues in black mass is still an unsolved challenge and needs to be addressed. The knowledge about these contents is crucial to evaluate if a black mass is suitable for the chosen recycling process. Electrolyte additives, such as vinylene carbonate or fluoroethylene carbonate, which are commonly used in state‐of‐the‐art LIBs are being categorized as potentially cancerogenic.^[^ ^15^, ^16^, ^17^, ^18^ ^]^ Lithium salts which are used in the electrolyte such as LiPF_6_ are acute toxic.^[^ ^19^, ^20^, ^21^ ^]^ During a recycling process, degradation products of higher toxicity categories, such as hydrofluoric acid or organofluorophosphates, can be formed.^[^ ^22^, ^23^ ^]^ These risks must be carefully considered to prevent damage to processing equipment, environmental contamination, and serious health hazards for the employees.

Another point for consideration is the value of the electrolyte compounds. In most cases, the recycling of the liquid electrolyte compounds is not yet cost effective. Never the less the lithium salts are the most valuable contents and are becoming of interest during the upcoming advances in LIB recycling.^[^ ^24^, ^25^, ^26^ ^]^ To reach the European Union (EU) recycling goal of 80 wt% of Li from LIB cells by 2031, this could be a necessary step enhancing the Li recovery rates.^[^ ^26^ ^]^ In addition, the EU regulations for the export of black mass which will demand more recycling activity inside of the EU instead of exporting it to states outside the union, while the contained phosphorous is named as critical raw material by the EU.^[^ ^27^, ^28^ ^]^

In this study we established a workflow for the extraction and quantitative investigation of electrolyte residues in black mass. Already developed LC methods, as by Schultz et al., were capable of quantifying some of the compounds of interest but still lacked compounds as ethylene glycol (EtGly).^[^ ^29^, ^30^ ^]^ Therefore, a high performance LC method hyphenated to a tandem mass spectrometer (HPLC‐MS) was developed and three different extraction methods were evaluated.

## 2 Experimental Section

### 2.1 Chemicals

Commercially available lithium ion battery recycling shred (black mass) gained from an industrial EV battery was used for the analysis. Hypergrade acetonitrile (ACN) was purchased from Merck KGaA (Darmstadt, Germany). Ultrapure water (H_2_O) was filtered with a Millipak 20 filter having a pore size of 0.22 μm that was installed into a Millipore Milli‐Q system (Bendford, USA). The organic carbonates dimethyl carbonate (DMC), diethyl carbonate (DEC), and ethylene carbonate (EC) were ordered from E‐Lyte (Münster, Germany). The 2,5‐dioxahexanedioicacid dimethyl ester (DMDOHC), and 2,5‐dioxahexanedioicacid diethyl ester (DEDOHC) were ordered from TCI Deutschland GmbH (Eschborn, Germany). EtGly, potassium hexafluorophosphate (99.5%), and sodium fluoride (99.9%) were ordered from Sigma‐Aldrich (Taufkirchen, Germany). Formic acid (98%–100%) was obtained from Merck KGaA (Darmstadt, Germany). MeOH Hypergrade LC‐MS (Merck KGaA, Darmstadt, Germany) was used as solvent for the extractions. Polytetrafluorethylene (PTFE) syringe filters (13 mm, 0.1 μm) (Dissolution Accessories, Oosterhout, The Netherlands) were used for sample preparation after the extractions. CaCl_2_ (98.0%) was ordered from Merck KGaA (Darmstadt, Germany).

### 2.2 Extractions

All extractions were performed using a ratio of 1 g black mass to 25 mL of solvent. Methanol (MeOH), ACN, and H_2_O were used. Twofold extractions were performed. All measurements were performed in triplicate for each extract. The presented data represent the average values with error bars indicating measurement uncertainties.

Shake extractions (SE) were performed using an advanced digital shaker (VWR International GmbH, Darmstadt, Germany) at 300 rpm and room temperature for 15 min. The extraction of the same black mas aliquot was performed three times, SE1, SE2, and SE3, to be able to evaluate the extraction efficacy of the first SE.

Ultrasonic assisted extractions (UAEs) were performed using an ultrasonic cleaning bath USC 300 T (VWR International GmbH, Darmstadt, Germany). The solvents were degassed using the ultrasonic bath previous to the extractions. In order to withstand a possible increase in pressure, screw‐on glass pressure pipes with a vent valve were used for the extraction. The black mass was suspended and then ultrasonicated at room temperature for 1 h.

Soxhlet extractions (SoxEs) were performed in a 30 mL Soxhlet apparatus using Cytiva Whatman extraction sleeves (Grade 603) (Fisher Scientific GmbH, Schwerte, Germany). All glass instruments were dried under reduced pressure at 60 °C. A drying tube filled with CaCl_2_ was used to prevent H_2_O intake from atmosphere. To prevent contamination of the extract with solid particles, the black mass was filled into the extraction sleeves and covered with dried glass wool. The solvents were heated until constant boiling could be achieved. For a better heat dissipation, glass beads were used.

All samples were centrifuged at 5000 rpm for 10 min using a Mega Star 600 R centrifuge (VWR International GmbH, Darmstadt, Germany). Afterward, the samples were filtered using a syringe filter, diluted in a mixture of H_2_O and ACN (95/5, %*v*/*v*) and a solution containing the internal standard dipropyl carbonate (DPC) was added. DPC was chosen because its chemical structure is similar to that of the other linear carbonates.

### 2.3 HPLC‐MS/MS Conditions

For the measurements, a HPLC system named UFLC prominence liquid chromatograph by Shimadzu (Nakagyo‐ku, Japan) hyphenated to a triple quadrupole MS (3200 QTRAPLC/MS/MS, AB Sciex, Darmstadt, Germany) was used. For ionization of the substances, electrospray ionization was used in the positive ion mode with an interface probe voltage of 5.5 kV. Polarity of the MS was set to positive. Curtain gas was set to 50 psi, collision gas to “High”, source temperature at 450 °C, ion source gas to 40 psi, drying gas to 50 psi, and the interface heater was turned on. A scheduled multiple reaction monitoring (sMRM) method was used for the analysis of organic carbonates, EtGly, and degradation products. The detection windows were set to 90 s and the target scan time to 1 s. Parameters and retention times for the analytes can be found in **Table** **1**.

**Table 1:** MS settings for the sMRM. The detection windows were set to 90 s and the target scan time to 1 s. Polarity of the MS was set to positive. Curtain gas was set to 50 psi, collision gas to “high”, ion spray voltage was set to 5500 V, source temperature at 450 °C, ion source gas to 40 psi, drying gas to 50 psi, and the interface heater was turned on (see supporting Info table S1, supporting information).

	Precursor ion/m/z	Quantifier ion/m/z	Ret. Time/min
EtGly	63.0	45.1	2.0
EC	89.0	45.1	3.0
DMC	91.1	63.1	7.0
EMC	105.2	77.1	9.7
DMDOHC	179.1	103.1	10.2
DEC	119.1	63.0	12.2
DEDOHC	207.1	89.2	13.9
DPC	147.2	41.2	16.2

The used eluent consisted of mobile phase A (aqueous 0.1 vol% formic acid solution for a more efficient ionization with 1 vol% ACN) and mobile phase B (ACN 0.1 vol% formic acid solution with 1 vol% H_2_O) with a flowrate of 0.5 mL min^−1^. The used gradient is described in the following: 0.0 min, 5% B; 1.9 min, 5% B; 14.0 min, 60% B; 16.0 min, 70% B; 16.9 min, 70% B; and 17.0 min, 5% B, 21 min, 5% B. The column oven was tempered to 40 °C. As stationary phase, a Restek Ultra Aqueous C18 column (Restek GmbH, Bad Homburg v. d. Höhe, Germany) with 15% carbon functionality was used. The dimensions were 3 mm × 150 mm, the pore size was 100 Å, and the particle size 3 μm. For the protection of the column, a Restek Trident Level 3 cartridge and filter pr‐column with an Ultra Aqueous C18 stationary phase was used. The dimension of the precolumn cartridge were 10 mm × 4 mm, the pore size was 1 Å with a particle size of 5 μm. The used filter had an inner diameter of 4 mm and a porosity of 2 μm.

The injection volume of the standards and samples was set to 5 μL. The autosampler was cooled to 15 °C to decrease evaporation. The LC system was controlled by LabSolutions (Version 5.65) (Shimadzu, Kyoto, Japan). The MS was controlled by Analyst software (1.5.2, Build 5704) (AB Sciex, Darmstadt, Germany).

### 2.4 IC‐CD Conditions

IC was performed on an 850 Professional IC (Metrohm, Herisau, Switzerland) with conductivity detection (CD). A Metrosep A Supp 7‐(250 × 4.0 mm, 5 μm; Metrohm) with a Metrosep A Supp 4/5 guard column was used for isocratic anion separation at 60 °C and a flow rate of 0.7 mL min^−1^ was applied. The developed method is based on Kraft et al. and further parameters and sample preparation were applied according to Henschel et al.^[^ ^31^, ^32^ ^]^

## 3 Results and Discussion

### 3.1 LC‐MS/MS Results

**Figure 1:**
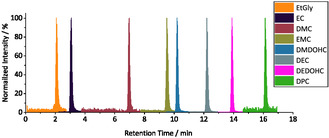
LC‐MS/MS chromatogram normalized to the individual maximum response at a concentration of 60 ppm (DPC 100 ppm). A representation of the absolute values can be found in the supporting information under Figure S1, Supporting Information.

To be able to analyze samples dissolved in H_2_O an LC separation had to be implemented instead of measurement via GC. By the use of the Restek Ultra Aqueous C_18_ reversed phase column a separation of the polar compounds EtGly and EC as well as the less polar compounds DEC and DEDOHC could be achieved with a complete runtime of only 21 min. As can be seen in **Figure** **1** a complete (baseline) separation of the chosen seven standard electrolyte compounds and degradation products as well as the internal standard could be achieved by the developed gradient. DPC was chosen due to the similar chemical structure to the other linear carbonates.

A triple quadrupole MS system was chosen for this study due to its ability to detect molecules with low mass‐to‐charge ratios (e.g., EtGly: 63.0 Da) and to perform tandem mass spectrometry (MS/MS). The sMRM method demonstrates high sensitivity for the selected analytes found in black mass extracts. The limits of detection (LOD) and limits of quantification (LOQ) values can be found in **Table** **2** determined in accordance with the regulations of DIN 32 645:2008‐11 with the calibration curve method. The LODs were determined to be in the sub‐ppm range, ranging from 0.04 to 0.51 ppm, while the LOQs were found to be in the range of 0.20 and 1.93 ppm (see Table 2).

**Table 2:** LOD and LOQ values of the analyzed electrolyte components using the LC‐MS/MS method. The LOD and LOQ are determined in accordance with the regulations of DIN 32 645:2008‐11 with the calibration curve method.

	EtGly	EC	DMC	EMC	DMDOHC	DEC	DEDOHC
LOD/ppm	0.51	0.20	0.39	0.24	0.04	0.12	0.16
LOQ/ppm	1.93	0.67	1.38	0.79	0.20	0.43	0.51

This LC‐MS/MS method enables the analysis of a broad spectrum of LIB electrolyte containing samples. Due to the polar end‐capped stationary phase not only nonpolar compounds can be separated. In comparison to commonly used GC systems no degradation of the stationary phase could be observed.^[^ ^9^ ^]^ Another application for this method is the fast analysis of aqueous wastewater from LIB production or recycling plants without the need for further sample preparation.

**Figure 2:**
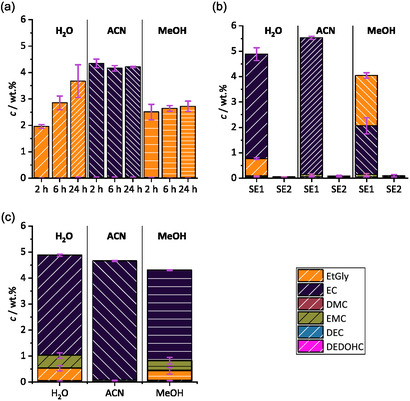
Overview of the LC‐MS/MS. Quantification results for the three solvents H_2_O, can, and MeOH: a) Stacked values of the Soxhlet extractions each solvent for 2 h, 6 h, and 24 h extraction times, b) stacked values of the first and second SE, and c) stacked values for the UAEs.

The extraction results (see **Figure** **2**) show strongly varying values depending on the applied solvent and extraction method. In total six analytes could be detected: EtGly, EC, DMC, ethyl methyl carbonate (EMC), DEC, and DEDOHC. For the SoxEs (see Figure 2a), ACN extracts show the highest overall content out of the black mass extracts and do not show variations beyond the standard deviations by increasing the extraction time from 2 to 24 h. These ACN extractions range between ≈4.1 wt% to 4.3 wt%. H_2_O shows an increase of the extraction over time but overall lower values than for ACN between 2.0 wt% and 3.7 wt%. MeOH extracts show constant values inside the standard deviations of the measurement. These values are lower than for ACN with values between 2.5 wt% and 2.7 wt%. It can be seen that ACN extracts mainly EC in contrast to H_2_O and MeOH in which mostly EtGly can be detected.

**Figure 3:**
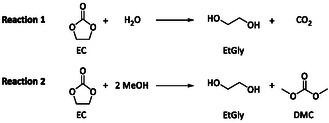
Reaction scheme of EC with the protic solvents 1 H_2_O and 2 MeOH. EtGly formation taken from Metzger et al. and DMC formation taken from Kim et al.^[^ ^33^, ^34^, ^35^ ^]^

This can be explained by the reaction of EC with the solvents as shown in **Figure** **3**.^[^ ^33^ ^]^ EC reacts with H_2_O forming EtGly and carbon dioxide. In an analog reaction, EC can also react with MeOH to form EtGly and DMC.^[^ ^29^ ^]^ The generated EtGly has a higher solubility in H_2_O and MeOH than EC leading to a higher extraction. Since ACN is an aprotic solvent, the generation of EtGly from EC is inhibited. If EtGly is measured, it has to be taken into account that this is a reaction product of EC. Thus, the initial value of EC in the black mass has to be calculated by the sum of the amount of substance of EC and EtGly. This formation is increased during the SoxEs in comparison to the SEs or UAEs which can be explained by the elevated temperatures and prolonged extraction times used for the SoxE. The results for the SEs (see Figure 2b) show overall higher extraction rates in comparison. These range between ≈3.9 wt% (MeOH) to 5.5 wt% (ACN). These extracts also show a higher relative concentration for EMC and DEC. It can also be seen that the extraction of EC and EtGly, respectively as reaction product, is almost complete in the first SE (SE1) approach since the SE2 shows only very small amounts of EC extraction. No results for SE3 are presented here, because no analytes could be detected in the extract.

**Figure 4:**
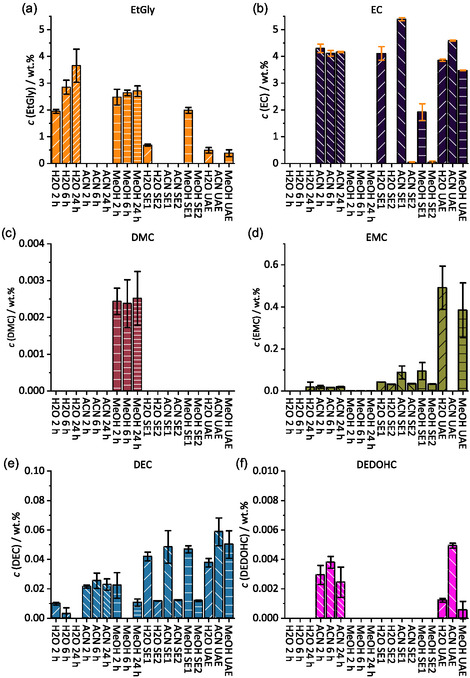
LC‐MS/MS quantification values for each analyte: a) EtGly, b) EC, c) DMC, d) EMC, e) DEC, and f) 2,5‐dioxahexanedioic acid diethyl ester (DEDOHC).

The results for the UAE (see Figure 2c) show lower concentrations than the SEs but overall higher concentrations than the SoxEs. Also, the concentrations of EtGly in H_2_O and MeOH are lower than for SE1. The concentration of EMC using H_2_O and MeOH shows to be the highest during the UAE extraction which can also be seen in **Figure** **4**d. This can be explained by the increased pressure and build‐up of cavitation bubbles inside the vials during the UAE. Thus, more EMC is extracted. During SoxEs, it is most likely that EMC is being evaporated due to the relatively low boiling point in comparison to the extraction solvents.^[^ ^34^ ^]^

In Figure 4c, the only extracts in which DMC is detected are the SoxE with the use of MeOH as solvents. This can also be explained by the reaction of MeOH with EC forming EtGly and DMC as by‐product. Since DMC has a low boiling point of 90 °C, it could not be detected in an equimolar concentration as EtGly.^[^ ^35^ ^]^ This also shows a limitation of this method: If DMC had been abundant in the black mass presented in this work, the extraction results with MeOH would have been overestimated and with other solvents underestimated. The extraction of DEC (see Figure 4e) shows comparable results in the range of the measurement error between SEs and UAEs of ≈0.04 wt% to 0.06 wt% using all solvents when adding up SE1 and SE2. DEDOHC (see Figure 4f) could only be found using ACN in all SoxEs and the UAE. Traces could be found in UAE using H_2_O and MeOH. Since DEDOHC is the least polar compound, this could be explained by the low solubility of DEDOHC in more polar solvents.

For the LC‐MS/MS results, it can be seen that ACN is most capable of extracting electrolyte compounds from the black mass without reacting with EC forming EtGly. The SE shows to be the most capable extraction method between the three analyzed, since it shows the highest overall extraction of organic electrolyte compounds from the black mass. Regarding extraction time, this method is with 15 min, in comparison to 1 h for the UAE and 2 h to 24 h for the SoxEs, the most favorable for a routine analysis of organic electrolyte residues in black mass.

### 3.2 IC‐CD Results

**Figure 5:**
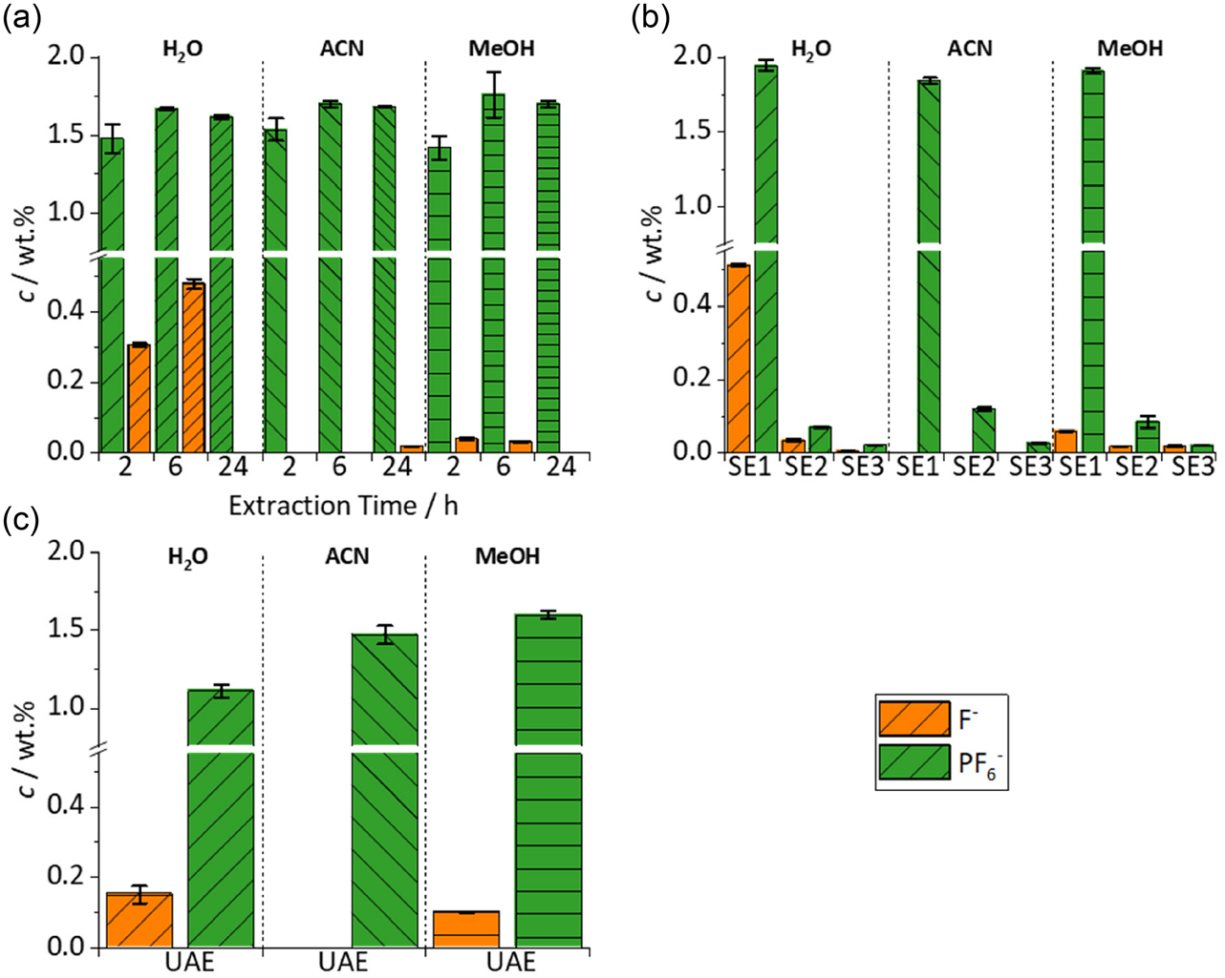
IC‐CD quantification results for the three solvents H_2_O, ACN, and MeOH. For each extraction three bars are shown for F^−^ and PF_6_^−^. a) Values of the Soxhlet extractions of each solvent for 2 h, 6 h, and 24 h extraction times. b) Values of the first and second SE. c) Values for the UAEs.

The results for the IC‐CD measurements of the extracts are shown in **Figure** **5**. Two analytes could be identified: F^−^ and PF_6_^−^. These extraction results do not show to be as heterogenic as the LC‐MS/MS results between the different extraction methods. In contrast to the organic electrolyte components, PF_6_^−^ is nonvolatile and equally soluble in the chosen solvents. This explains that findings for SoxEs and SEs are in a comparable range of 1.5 wt% to 2.0 wt%. For UAEs the extraction results are lower with values varying between 1.1 wt% to 1.6 wt%. The result for the extraction from PF_6_^−^ using H_2_O is nearly doubled from 1.11 wt% for UAE to 1.95 wt% when using the SE. This shows that the movement induced by shaking the sample as well as the multiple extraction over time during SoxE helps extracting the PF_6_^−^. Extracting using ACN and MeOH shows similar results for the SE with 1.84 wt% and 1.90 wt%, respectively. For all SoxEs, the extraction of PF_6_^−^ is increased from 2 to 6 h. For ACN and MeOH, the extracted PF_6_^−^ values stay approximately the same from 6 to 24 h.

These results show that black mass contains high concentrations of LIB electrolyte salt. In principal, this could be a chance for recycling industry to utilize black mass for a sustainable production of this fluor containing valuable resource.^[^ ^36^, ^37^ ^]^ To add up, this salt content could complicate recycling steps as the flotation.^[^ ^38^ ^]^

F^−^ is in the range of 0.0 wt% to 0.5 wt%. Extraction with H_2_O shows high concentrations for F^−^ with 0.51 wt% for SE. In contrast, using MeOH, only 0.02 wt% was extracted and with ACN no F^−^could be detected. This can be explained by the PF_6_^−^ degradation reacting with protic solvents to HF.^[^ ^39^ ^]^ This is most prominent in H_2_O, less in MeOH, and cannot be observed using ACN since the reaction cannot occur with this aprotic solvent.^[^ ^40^ ^]^ This degradation reaction seems to be occurring the fastest in the SE using H_2_O. In contrast to that during the SoxEs, the concentration of F^−^ is increasing from 2 over 6 to 24 h to reach a comparable level. Since no degradation of PF_6_^−^ in reaction with ACN is literature known, this value can be set as the baseline F^−^ concentration of the black mass.

To conclude the results gained by IC‐CD show that SEs in total are again the most time efficient and reproducible way to extract the conducting salt and its degradation product F^−^ from black mass. The highest extraction could be achieved by using H_2_O extracting ≈1.95 wt% PF_6_^−^ and 0.51 wt% F^−^ totaling up to 2.46 wt% of anionic species. This could be explained by two differing effects. The first one is the high solubility of F^−^ in H_2_O in contrast to its low solubility in MeOH and ACN.^[^ ^41^, ^42^ ^]^ On the other hand, this could imply that F^−^ in the form of HF is part of the black mass and can be extracted well using H_2_O.^[^ ^43^ ^]^

H_2_O was chosen due to two points: its high dipole moment enables good solvation for the investigated ionic species (PF_6_^−^ and F^−^) and H_2_O is industrial practice for hydrometallurgical recycling processes thus enabling to mimic the conditions during black mass leaching.^[^ ^44^ ^]^ However, employing H_2_O as extraction solvent depicts the inevitable process of HF generation during leaching of PF_6_^−^ containing black mass.^[^ ^45^, ^46^ ^]^ In a laboratory scale, the F^−^ concentration is in a carefully manageable range (here up to 5 mg F^−^ per extraction), but it should be noted that the evolution of HF has to be addressed during the treatment of the process H_2_O and high safety standards have to be maintained during black mass leaching.^[^ ^23^ ^]^ The formation and release of HF require rigorous safety standards and process H_2_O treatment to mitigate risks to worker and the environment. Furthermore, F^−^ has been associated with potential neurological disorders at low levels.^[^ ^47^, ^48^ ^]^ To set this in perspective: Reported by the European food safety authority, the adequate daily fluoride intake is 50 μg kg^−1^.^[^ ^49^, ^50^, ^51^ ^]^ In a hydrometallurgical recycling plant where tons of black mass are extracted, this can impose a severe risk for employees or pollution of the environment and H_2_O supply. Therefore in Germany a concentration of 20 mg L^−1^ F^−^ has to be undercut.^[^ ^52^ ^]^

## 4 Conclusions and Outlook

In this study, a comprehensive work flow for quality control of black mass during the recycling process could be presented. Therefore, three different extraction methods (Soxhlet, ultrasonic assisted, and SE) were compared using three solvents (MeOH, ACN, and H_2_O). In order to analyze these extracts, a LC method was developed. This method is capable of separating the most common LIB electrolyte components EC, DMC, EMC, DEC as well as the degradation products EtGly, DMDOHC, and DEDOHC and an internal standard, DPC.^[^ ^10^, ^53^ ^]^ The analytes could be identified and quantified using a sMRM method performed by a triple quadrupole MS. This combination is enabling the analysis of aqueous solutions, and the simultaneous analysis of polar compounds as EtGly and less polar compounds as DEC in the same measurement. For the quantification of the anion of the conducting salt, PF_6_^−^ and the degradation product F^−^, an already established method by using IC hyphenated to a CD was utilized.^[^ ^9^, ^30^ ^]^ Additionally, these extractions show hydrolysis which are potentially observed during an aqueous recycling process.

Our results indicate that SE using ACN yields reliable quantification of less volatile organic compounds (EC, DEC, and DEDOHC) and PF_6_^−^ anions without inducing noticeable decomposition of the analytes by solvolysis. In contrast both H_2_O and MeOH show decomposition products of EC forming EtGly and DMC, respectively. The UAE shows a high capability of extracting the volatile compound EMC due to the increased pressure build‐up of cavitation bubbles inside the vials during the extraction. SoxE shows the least favorable extraction results. The values are lower than the results for SE and show higher degradation by introducing thermal stress to the PF_6_^−^ and EC. The results for SoxE can be used to anticipate the influence of thermal stress during hydrometallurgical recycling of black mass.

Due to the lack of solubility of F^−^ in ACN, only MeOH and H_2_O can be used as an extraction solvent for F^−^ quantification. H_2_O shows high extractions which can be explained by the hydrolysis of the conducting salt PF_6_^−^, while MeOH shows lower concentrations, which could be the real values of F^−^ in the black mass. No single solvent can be universally recommended, but the choice should be based on the specific analytical requirements. ACN is best suitable for the analysis of the organic compounds, while MeOH shows the highest extraction results for ionic compounds without leading to PF_6_^−^ degradation.

To further advance this LC method, it would be possible to raise the number of analytes (e.g., organofluorophosphates, electrolyte additives) that can be separated and quantified by adjusting the used gradient or by introducing a second separative dimension. A solid sampling technique, e.g., thermal desorption‐GC‐MS, would also be a future alternative, skipping the extraction step and thus reducing the amount of time, complexity, and labor required. The flexibility of the LC‐MS/MS method, capable of analyzing H_2_O samples and samples dissolved in organic solvents, enabling quantification of electrolyte compounds in lots of use cases, e.g., industrial recycling plant wastewater.

## Conflict of Interest

The authors declare no conflict of interest.

## Supporting information

Supplementary Material

## Data Availability

The data that support the findings of this study are available on request from the corresponding author. The data are not publicly available due to privacy or ethical restrictions.

## References

[1] J. Neumann , M. Petranikova , M. Meeus , J. D. Gamarra , R. Younesi , M. Winter , S. Nowak , Adv. Energy Mater. 2022, 12, 2102917.

[2] C. Xu , Q. Dai , L. Gaines , M. Hu , A. Tukker , B. Steubing , Commun. Mater. 2020, 1, 1.

[3] G. A. Campbell , Miner. Econ. 2019, 33, 21.

[4] C. Banza Lubaba Nkulu , L. Casas , V. Haufroid , T. de Putter , N. D. Saenen , T. Kayembe‐Kitenge , P. Musa Obadia , D. Kyanika Wa Mukoma , J.‐M. Lunda Ilunga , T. S. Nawrot , O. Luboya Numbi , E. Smolders , B. Nemery , Nat. Sustain. 2018, 1, 495. 30288453 10.1038/s41893-018-0139-4 PMC6166862

[5] M. Grützke , S. Krüger , V. Kraft , B. Vortmann , S. Rothermel , M. Winter , S. Nowak , ChemSusChem 2015, 8, 3433. 26360935 10.1002/cssc.201500920

[6] C. Peschel , S. van Wickeren , Y. Preibisch , V. Naber , D. Werner , L. Frankenstein , F. Horsthemke , U. Peuker , M. Winter , S. Nowak , Chemistry 2022, 28, e202200485. 35188309 10.1002/chem.202200485 PMC9311206

[7] M. Dommaschk , T. Sieber , J. Acker , J. Anal. At. Spectrom. 2024, 39, 2522.

[8] P. Bieker , M. Winter , Chemie Unserer Zeit 2016, 50, 172.

[9] V. Kraft , W. Weber , M. Grützke , M. Winter , S. Nowak , RSC Adv. 2015, 5, 80150.

[10] J. Henschel , C. Peschel , S. Klein , F. Horsthemke , M. Winter , S. Nowak , Angew. Chem. Int. Ed. Engl. 2020, 59, 6128. 32012404 10.1002/anie.202000727 PMC7187180

[11] Y. Liao , H. Zhang , Y. Peng , Y. Hu , J. Liang , Z. Gong , Y. Wei , Y. Yang , Adv. Energy Mater. 2024, 14, 2304295.

[12] J. S. Edge , S. O’Kane , R. Prosser , N. D. Kirkaldy , A. N. Patel , A. Hales , A. Ghosh , W. Ai , J. Chen , J. Yang , S. Li , M.‐C. Pang , L. Bravo Diaz , A. Tomaszewska , M. W. Marzook , K. N. Radhakrishnan , H. Wang , Y. Patel , B. Wu , G. J. Offer , Phys. Chem. Chem. Phys. 2021, 23, 8200. 33875989 10.1039/d1cp00359c

[13] C. Schultz , S. Vedder , B. Streipert , M. Winter , S. Nowak , RSC Adv. 2017, 7, 27853.

[14] V. Kraft , W. Weber , B. Streipert , R. Wagner , C. Schultz , M. Winter , S. Nowak , RSC Adv. 2016, 6, 8.

[15] X. Liu , M. Zhang , R. Ping , Y. Chen , X. Hu , J. Power Sources 2025, 649, 237428.

[16] “Vinylencarbonat contains ≤2% BHT as stabilizer, 97% | Sigma‐Aldrich”, to be found under https://www.sigmaaldrich.com/DE/de/product/aldrich/v2607 2025 (accessed: April 2025).

[17] P. Janssen , R. Schmitz , R. Müller , P. Isken , A. Lex‐Balducci , C. Schreiner , M. Winter , I. Cekić‐Lasković , R. Schmitz , Electrochim. Acta 2014, 125, 101.

[18] “1,3,2‐Dioxathiolan‐2,2‐dioxid 98% | Sigma‐Aldrich”, to be found under https://www.sigmaaldrich.com/DE/de/product/aldrich/471690?srsltid=AfmBOoqj1O7B6‐bpj2enOjoY5_6D93DpXPiWOAU0OT_AXTJ02‐3fgbjL 2025 (accessed: April 2025).

[19] P. Bieker , M. Winter , Chemie Unserer Zeit 2016, 50, 26.

[20] “GESTIS‐Stoffdatenbank: Lithium‐bis(trifluormethylsulfonyl”, to be found under https://gestis.dguv.de/data?name=901060 2024 (accessed: April 2025).

[21] “GESTIS‐Stoffdatenbank: Lithiumhexafluorophosphat”, to be found under https://gestis.dguv.de/data?name=132948 2024 (accessed: April 2025).

[22] A. Rensmo , E. K. Savvidou , I. T. Cousins , X. Hu , S. Schellenberger , J. P. Benskin , Environ. Sci. Process. Impacts 2023, 25, 1015. 37195252 10.1039/d2em00511e

[23] “GESTIS‐Stoffdatenbank: Fluorwasserstoff”, to be found under https://gestis.dguv.de/data?name=001040 2024 (accessed: April 2025).

[24] B. Niu , Z. Xu , J. Xiao , Y. Qin , Chem. Rev. 2023, 123, 8718. 37339582 10.1021/acs.chemrev.3c00174

[25] “Verordnung ‐ 2023/1542 ‐ EN ‐ EUR‐Lex”, to be found under https://eur‐lex.europa.eu/legal‐content/DE/TXT/?uri=celex:32023R1542 2025 (accessed: April 2025).

[26] S. Nowak , M. Winter , Molecules 2017, 22, 403. 28272327 10.3390/molecules22030403 PMC6155197

[27] Internal Market, Industry, Entrepreneurship and SMEs, “Critical raw materials”, to be found under https://single‐market‐economy.ec.europa.eu/sectors/raw‐materials/areas‐specific‐interest/critical‐raw‐materials_en 2025 (accessed: April 2025).

[28] Environment, “Battery‐related waste codes update set to boost circular economy”, to be found under https://environment.ec.europa.eu/news/battery‐related‐waste‐codes‐update‐set‐boost‐circular‐economy‐2025‐03‐05_en 2025 (accessed: April 2025).

[29] D. Kim , M. Lee , Y. Shin , J. Lee , J. W. Lee , Chem. Eng. Process. Process Intensification 2023, 192, 109519.

[30] C. Schultz , V. Kraft , M. Pyschik , S. Weber , F. Schappacher , M. Winter , S. Nowak , J. Electrochem. Soc. 2015, 162, A629.

[31] V. Kraft , M. Grützke , W. Weber , M. Winter , S. Nowak , J. Chromatogr. A 2014, 1354, 92. 24939088 10.1016/j.chroma.2014.05.066

[32] J. Henschel , F. Horsthemke , Y. P. Stenzel , M. Evertz , S. Girod , C. Lürenbaum , K. Kösters , S. Wiemers‐Meyer , M. Winter , S. Nowak , J. Power Sources 2020, 447, 227370.

[33] M. Metzger , B. Strehle , S. Solchenbach , H. A. Gasteiger , J. Electrochem. Soc. 2016, 163, A1219.

[34] “Ethylmethylcarbonat 99% | Sigma‐Aldrich”, to be found under https://www.sigmaaldrich.com/DE/de/product/aldrich/754935 2025 (accessed: April 2025).

[35] “Dimethylcarbonat MSDS ‐ 803525 ‐ Merck”, to be found under https://www.merckmillipore.com/DE/de/product/msds/MDA_CHEM‐803525?Origin=PDP 2025 (accessed: April 2025).

[36] J. Wang , X. Cui , L. Song , J. Zhu , Y. Wang , F. Zong , N. Zhang , D. Zhao , S. Li , Green Chem. 2024, 26, 2162.

[37] “Lithium Hexafluorophosphate Prices, News and Demand”, to be found under https://www.imarcgroup.com/lithium‐hexafluorophosphate‐pricing‐report?utm_source=chatgpt.com (accessed: April 2025).

[38] A. M. Salces , I. Bremerstein , M. Rudolph , A. Vanderbruggen , Miner. Eng. 2022, 184, 107670.

[39] L. Terborg , S. Nowak , S. Passerini , M. Winter , U. Karst , P. R. Haddad , P. N. Nesterenko , Anal. Chim. Acta 2012, 714, 121. 22244145 10.1016/j.aca.2011.11.056

[40] K. O. Christe , W. W. Wilson , J. Fluorine Chem. 1990, 47, 117.

[41] D. A. Wynn , M. M. Roth , B. D. Pollard , Talanta 1984, 31, 1036. 18963717 10.1016/0039-9140(84)80244-1

[42] “Hydrogen Fluoride (HF) | Toxic Substances | Toxic Substance Portal | ATSDR”, to be found under https://wwwn.cdc.gov/TSP/substances/ToxSubstance.aspx?toxid=250 2025 (accessed: April 2025).

[43] S. Wiemers‐Meyer , M. Winter , S. Nowak , Phys. Chem. Chem. Phys. 2016, 18, 26595. 27711648 10.1039/c6cp05276b

[44] M. Bhar , S. Ghosh , S. Krishnamurthy , Y. Kaliprasad , S. K. Martha , RSC Sustainability 2023, 1, 1150.

[45] M. Stich , M. Göttlinger , M. Kurniawan , U. Schmidt , A. Bund , J. Phys. Chem. C 2018, 122, 8836.

[46] S. Solchenbach , M. Metzger , M. Egawa , H. Beyer , H. A. Gasteiger , J. Electrochem. Soc. 2018, 165, A3022.

[47] K. W. Taylor , S. E. Eftim , C. A. Sibrizzi , R. B. Blain , K. Magnuson , P. A. Hartman , A. A. Rooney , J. R. Bucher , JAMA Pediatr. 2025, 179, 282. 39761023 10.1001/jamapediatrics.2024.5542 PMC11877182

[48] G. H. N. Miranda , M. O. P. Alvarenga , M. K. M. Ferreira , B. Puty , L. O. Bittencourt , N. C. F. Fagundes , J. P. Pessan , M. A. R. Buzalaf , R. R. Lima , Sci. Rep. 2021, 11, 22659. 34811523 10.1038/s41598-021-99688-w PMC8609002

[49] Scientific Opinion on Dietary Reference Values for fluoride, EFSA Journal 2013, 11, 3332. 10.2903/j.efsa.2013.3005 PMC13159830 42124964

[50] Panel (EFSA Panel on Dietetic Products, Nutrition and Allergies), 2013. Scientific opinion on Dietary Reference Values for fluoride. EFSA Journal 2013; 11 …, **2013**.

[51] S. Guth , S. Hüser , A. Roth , G. Degen , P. Diel , K. Edlund , G. Eisenbrand , K.‐H. Engel , B. Epe , T. Grune , V. Heinz , T. Henle , H.‐U. Humpf , H. Jäger , H.‐G. Joost , S. E. Kulling , A. Lampen , A. Mally , R. Marchan , D. Marko , E. Mühle , M. A. Nitsche , E. Röhrdanz , R. Stadler , C. van Thriel , S. Vieths , R. F. Vogel , E. Wascher , C. Watzl , U. Nöthlings , J. G. Hengstler , Arch. Toxicol. 2020, 94, 1375. 32382957 10.1007/s00204-020-02725-2 PMC7261729

[52] “Anhang 40 AbwV ‐ Einzelnorm”, to be found under https://www.gesetze‐im‐internet.de/abwv/anhang_40.html 2025 (accessed: July 2025).

[53] H. Yoshida , T. Fukunaga , T. Hazama , M. Terasaki , M. Mizutani , M. Yamachi , J. Power Sources 1997, 68, 311.

