# Supplementary material for: Uncovering the Residual Electrolyte Quantity in Recycled Battery Black Mass via Liquid Chromatography Tandem Mass Spectrometry and Ion Chromatography‐Conductivity Detection
Source: ChemSusChem. 2025 Aug 18;18(19):e202501158. doi: 10.1002/cssc.202501158 (PMC12487738; doi:10.1002/cssc.202501158)
Supplement: Supplementary file 1 — Supplementary Material [file CSSC-18-e202501158-s001.pdf]

Uncovering the Residual Electrolyte Quantity in Recycled Battery Black Mass *via* LC-MS/MS and IC-CD

Jakob Michael Hesper[a], Simon Weigel[b], Jan Henrik Hintemann[a], Jaroslav Minář[a], Martin Winter[a,c], Simon Wiemers-Meyer[a] and Sascha Nowak[a,*]

[a] J.M. Hesper, J.H. Hintemann, Dr. J. Minář, Prof. M. Winter, Dr. S. Wiemers-Meyer, Dr. S. Nowak
MEET Battery Research Center
University of Münster
Corrensstraße 46, 48149 Münster, Germany
E-mail: sascha.nowak@uni-muenster.de; m.winter@fz-juelich.de
[b] Dr. S. Weigel
Königswarter & Ebell Chemische Fabrik GmbH
Im Ennepetal 19-21, 58135 Hagen, Germany
[c] Prof. M. Winter
Helmholtz-Institute Münster, IMD-4
Forschungszentrum Jülich
Corrensstraße 46, 48149 Münster, Germany

Supporting Info

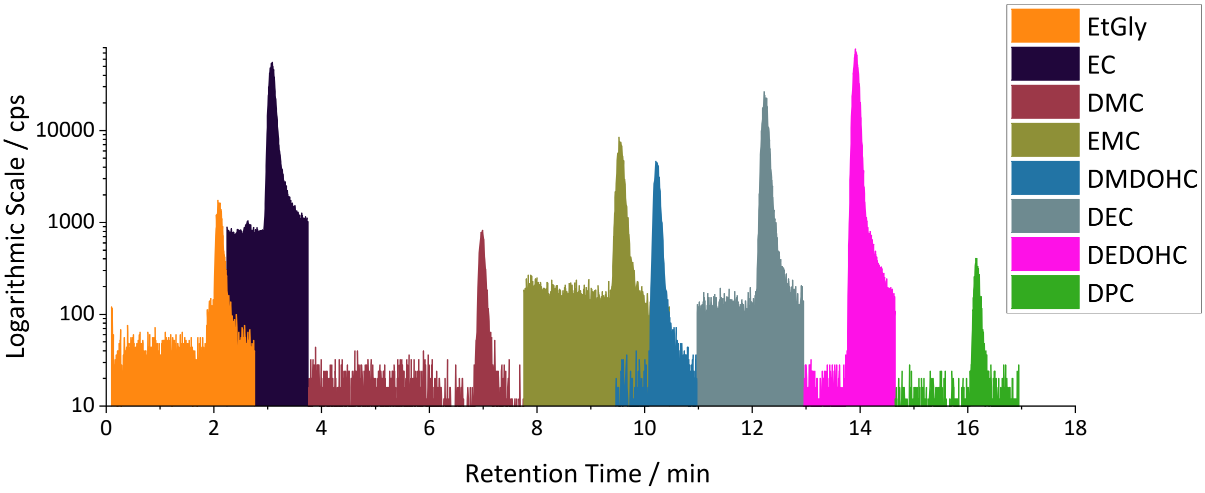

Figure S 1: Logarithmic scale of the absolute intensity for the MRM detection.

Table S 1. Source and mass spectrometer parameter for the analytes.

ID	Precursor ion / *m/z*	Quantifier ion/ *m/z*	Ret. Time / min	Declustering Potential / V	Entrance Potential / V	Collision Cell Entrance Potential / V	Collision Cell Exit Potential / V
EC	89.0	45.1	3.0	32.0	9.0	11.0	20.0
DMC	91.1	63.1	7.0	22.0	7.0	13.0	12.0
EMC	105.2	77.1	9.7	11.0	5.0	12.0	17.0
DEC	119.1	63.0	12.2	10.0	5.0	11.0	16.0
DMDOHC	179.1	103.1	10.2	30.0	7.0	12.5	17.0
DEDOHC	207.1	89.2	13.9	20.0	6.0	13.0	22.0
VEC	115.0	39.2	7.0	15.0	5.0	20.0	45.0
EtGly	63.0	45.1	2.0	25.0	10.0	13.0	17.0
DPC	147.2	41.2	16.2	25.0	10.0	13.7	50.0

Table S 2. Experimental parameters of the optimized LC-MS/MS method.

Instrumental Parameter	Value
**Prominence UFLC**	
Binary pumps	LC-20AD
Degasser	DGU-20A3
Autosampler	SIL-20AC
Column oven	CTO-20AC
Eluent	Water/Acetonitrile
Flow rate	0.5 mL min^{-1}
Injection volume	5 µL
**Triple quadrupole 3200 QTRAP® MS**	
Curtain gas / psi	50
Ion spray voltage / V	5500
Temperature / °C	450
Ion source gas 1 / psi	40
Ion source gas 2 / psi	50
Interface heater	On
Scan time / s	0.1
